# Feasibility of a Home-Based Exercise Program for Managing Posttransplant Metabolic Syndrome in Lung and Liver Transplant Recipients: Protocol for a Pilot Randomized Controlled Trial

**DOI:** 10.2196/35700

**Published:** 2022-03-23

**Authors:** Dmitry Rozenberg, Daniel Santa Mina, Sahar Nourouzpour, Encarna Camacho Perez, Brooke Lyn Stewart, Lisa Wickerson, Cynthia Tsien, Nazia Selzner, Josh Shore, Meghan Aversa, Minna Woo, Sandra Holdsworth, Karina Prevost, Jeff Park, Amirhossein Azhie, Ella Huszti, Elizabeth McLeod, Sarah Dales, Mamatha Bhat

**Affiliations:** 1 Respirology and Lung Transplantation, Temerty Faculty of Medicine Toronto General Hospital Research Institute University Health Network Toronto, ON Canada; 2 Ajmera Transplant Program University Health Network Toronto, ON Canada; 3 Faculty of Kinesiology and Physical Education University of Toronto Toronto, ON Canada; 4 Department of Anesthesia and Pain Management University Health Network Toronto, ON Canada; 5 GoodHope Ehlers-Danlos Syndrome Program University Health Network Toronto, ON Canada; 6 Nutrition Ajmera Transplant Centre University Health Network Toronto, ON Canada; 7 Department of Physical Therapy University of Toronto Toronto, ON Canada; 8 Gastroenterology and Liver Transplantation Temerty Faculty of Medicine University Health Network Toronto, ON Canada; 9 Endocrinology, Temerty Faculty of Medicine Toronto General Hospital Research Institute University Health Network Toronto, ON Canada; 10 Canadian Donation and Transplantation Research Program Edmonton, ON Canada; 11 Biostatistics Research Unit University Health Network Toronto, ON Canada

**Keywords:** lung transplant, liver transplant, posttransplant metabolic syndrome, exercise training, randomized controlled trial, pilot study

## Abstract

**Background:**

Posttransplant metabolic syndrome (PTMS) is a common contributor to morbidity and mortality among solid organ transplant recipients in the late posttransplant period (≥1 year). Patients diagnosed with PTMS are at a higher risk of cardiovascular disease and frequently experience decreased physical function and health-related quality of life (HRQL). Studies in the early posttransplant period (<1 year) have shown the benefits of facility-based exercise training on physical function and HRQL, but have not evaluated the effects on metabolic risk factors. It remains unclear whether home-based exercise programs are feasible and can be delivered at a sufficient exercise dose to have effects on PTMS. This protocol outlines the methodology of a randomized controlled trial of a partly supervised home-based exercise program in lung transplant (LTx) and orthotopic liver transplant (OLT) recipients.

**Objective:**

This study aims to evaluate the feasibility (ie, recruitment rate, program adherence, attrition, safety, and participant satisfaction) of a 12-week individualized, home-based aerobic and resistance training program in LTx and OLT recipients initiated 12 to 18 months after transplantation, and to assess estimates of intervention efficacy on metabolic risk factors, exercise self-efficacy, and HRQL.

**Methods:**

In total, 20 LTx and 20 OLT recipients with ≥2 cardiometabolic risk factors at 12 to 18 months after transplantation will be randomized to an intervention (home-based exercise training) or control group. The intervention group will receive an individualized exercise prescription comprising aerobic and resistance training, 3 to 5 times a week for 12 weeks. Participants will meet on a weekly basis (via videoconference) with a qualified exercise professional who will supervise exercise progression, provide support, and support exercise self-efficacy. Participants in both study groups will receive a counseling session on healthy eating with a dietitian at the beginning of the intervention. For the primary aim, feasibility will be assessed through recruitment rate, program adherence, satisfaction, attrition, and safety parameters. Secondary outcomes will be measured at baseline and 12 weeks, including assessments of metabolic risk factors (ie, insulin resistance, abdominal obesity, blood pressure, and cholesterol), HRQL, and exercise self-efficacy. Descriptive statistics will be used to summarize program feasibility and effect estimates (means and 95% CIs) for sample size calculations in future trials.

**Results:**

Enrollment started in July 2021. It is estimated that the study period will be 18 months, with data collection to be completed by December 2022.

**Conclusions:**

A partly supervised home-based, individually tailored exercise program that promotes aerobic and resistance training and exercise self-efficacy may be an important intervention for improving the metabolic profile of LTx and OLT recipients with cardiometabolic risk factors. Thus, characterizing the feasibility and effect estimates of home-based exercise constitutes the first step in developing future clinical trials designed to reduce the high morbidity associated with PTMS.

**Trial Registration:**

ClinicalTrials.gov NCT04965142; https://clinicaltrials.gov/ct2/show/NCT04965142

**International Registered Report Identifier (IRRID):**

DERR1-10.2196/35700

## Introduction

### Background

Posttransplant metabolic syndrome (PTMS) is prevalent among solid organ transplant recipients with >25% of lung transplant (LTx) and 50% of orthotopic liver transplant (OLT) recipients developing PTMS within 12-18 months [1,2]. PTMS comprises impaired glucose tolerance, obesity, hypercholesterolemia, and hypertension and has been associated with an increased risk of hospital readmissions and cardiovascular morbidity accompanied by decreased long-term survival [3-5]. LTx and OLT recipients frequently present with risk factors for PTMS, including increased appetite and weight gain along with a reversal of the pretransplant catabolic state, immunosuppression, and physical inactivity [6]. Our research group has shown that in a sample of >2000 OLT recipients in the Toronto Liver Program (1990-2015), 35% had pre-existing, new onset, or transient posttransplant diabetes mellitus, a PTMS risk factor associated with decreased long-term survival [7]. A retrospective evaluation of 227 LTx recipients conducted by our group also demonstrated a significant increase in the prevalence of metabolic risk factors (hypertension, hyperlipidemia, diabetes mellitus, and obesity) by 1 year after transplantation (≥3 factors: 26%) compared with before transplantation (11%) [8].

LTx and OLT recipients have been observed to engage in less physical activity than the general population and report barriers to exercise, including lack of specific exercise guidelines, medication side effects, comorbidities, and inability to access fitness facilities [9,10]. In a cross-sectional survey of 656 solid organ transplant recipients, van Adrichem et al [11] found that only 56% of patients met the physical activity guidelines and engaged in ≥150 minutes per week of moderate aerobic activity after transplantation. A 2019 consensus statement [12] highlighted the importance of strategies to mitigate posttransplant metabolic risk factors with physical training. The recommendation for solid organ transplant recipients is to practice moderate to vigorous intensity physical training (either aerobic or aerobic plus resistance training) 3 to 5 times a week for a minimum of 8 weeks [12].

The posttransplant period offers an opportunity for transplant recipients to derive significant metabolic and functional benefits from exercise, as most of them can train at a greater volume and intensity than in the pretransplant period [13]. In this regard, 24 sessions of 30-minute treadmill exercise sessions were associated with a significant increase in resting energy expenditure in OLT recipients [14]. Moreover, a meta-analysis of 15 randomized controlled trials (RCTs) showed a significant reduction in body fat percentage in OLT recipients (1 trial, 119 patients) who participated in physical exercise training (median −5.40%, 95% CI −8.03% to −2.77%; P<.001) [15]. Most of the exercise programs included in this meta-analysis implemented exercise interventions starting a year after transplantation (program duration between 8 and 24 weeks) and focused on aerobic and strength training.

To date, most exercise interventions in LTx and OLT recipients have been delivered as supervised, facility-based programs within the first 12 months after transplantation [16-19]. However, facility-based programs present challenges for patients [12,20], especially in the late posttransplant period. Given the decreased frequency of clinic visits, relocation requirements, return to work [21], and the interruption of hospital and community-based programs during the COVID-19 pandemic [22], home-based exercise programs can overcome some of these challenges and have been recognized as an important strategy that requires further research [20].

In light of the current challenges to health care delivery because of the COVID-19 pandemic, telerehabilitation and remote exercise interventions are gaining attention as the preferred modalities for promoting physical activity and exercise behaviors among solid organ transplant recipients [23-26]. Only a few studies on home-based exercise programming in the early and late posttransplant periods have been conducted in both LTx [27-29] and OLT [30,31] recipients, and they have suggested benefits to aerobic capacity, quadriceps strength, and health-related quality of life (HRQL). In one of these studies, program adherence in OLT recipients was poor (only 37% of participants completed ≥50% of exercises with bimonthly phone calls) [30], whereas adherence was not assessed in a home-based program with 12 LTx recipients (mean 36 months, SD 33 months after transplantation) [27]. Furthermore, the effects of home-based training on metabolic risks were evaluated in only 1 OLT study, which used a combination of a few personalized sessions and group telehealth classes for exercise and nutritional counseling for a 3-month period and showed a modest improvement in metabolic syndrome and HRQL [32]. However, the optimal structure of counseling, exercise prescription, and effects on metabolic risk factors among solid organ transplant groups remains to be defined [12].

There is limited literature surrounding the experiences of transplant recipients concerning knowledge of, motivation for, and barriers to exercise training, which limits our understanding of the feasibility of real-world implementation. Several strategies to engage participants in exercise interventions and optimize adherence have been explored for chronic diseases [9]. The literature on chronic disease management highlights that adherence can be increased by providing close support by an exercise professional, simplifying the number of exercises, and fostering self-efficacy for exercise [33]. The support and coaching by the exercise professional can be enhanced with the use of telecommunication and the ability of the participants to readily contact the health coach as needed [26,27]. In addition, focusing on four sources of self-efficacy built into an exercise program (mastery, vicarious experiences, verbal feedback, physiological and emotional state) has been shown to significantly improve exercise adherence in patients with cardiometabolic risk factors [34,35] but has not been applied to transplant recipients.

These gaps in knowledge are important to address as optimal effects on PTMS are likely achieved if exercise is performed with sufficient volume (at least moderate intensity, accumulating ≥150 min/week) [36]. However, it remains unclear whether this training dose is optimal or if it can be achieved with a home-based program targeting metabolic risk factors starting at 12-18 months after transplantation, a critical period prognostic of long-term PTMS and cardiovascular morbidity [37]. Before these efficacy questions can be addressed with an appropriately powered RCT, a pilot study evaluating the feasibility of a home-based exercise program is needed.

### Aims

The specific aims of this study are (1) to evaluate the feasibility of a 12-week individualized, home-based aerobic and resistance training program in LTx and OLT recipients at 12-18 months after transplantation and (2) to assess estimates of intervention efficacy on PTMS risk factors, exercise self-efficacy, and HRQL. We hypothesize that we will achieve a recruitment rate of 30% of eligible LTx and OLT recipients, participants in the intervention group will demonstrate an adherence rate of ≥70% to the prescribed exercise dose (including attainment of the prescribed exercise progression over time) and that PTMS risk factors, self-efficacy, and HRQL will be improved in the intervention group compared with the control group.

## Methods

### Design

This paper describes a pilot RCT to assess the feasibility of a phase 3 RCT examining the effects of an individualized, partly supervised, home-based exercise intervention in LTx and OLT recipients. The study is being conducted at the Toronto General Hospital in Toronto, Canada, and ethical approval was obtained from the research ethics board at the University Health Network (study ID: 20-5185). The study was registered at ClinicalTrials.gov NCT04965142. Informed written consent will be obtained from all participants before starting any research activities.

This protocol follows the SPIRIT (Standard Protocol Items: Recommendations for Interventional Trials) guidelines [38], the CONSORT (Consolidated Standards of Reporting Trials) statement [39], and the Consensus on Exercise Reporting Template [40]. Two patient partners are part of the research team, have provided input into the study design, and will contribute support and their lived experience throughout the study. The research team will hold meetings with patient partners every 3 to 6 months to collect feedback on study progress, assess concerns, and work on knowledge translation tools once the study is completed. Modifications to the study protocol will be approved by the research ethics board before implementation, communicated to funding agencies, and outlined in future publications.

### Participants

The study sample will consist of adult LTx and OLT recipients (≥18 years) with at least two or more metabolic risk factors (ie, hypertension, hyperlipidemia, diabetes, and obesity) at 12 to 18 months after transplantation. Exclusion criteria include (1) active cardiovascular disease (eg, recent heart attack, significant coronary artery disease on cardiac catheterization, heart failure, uncontrolled arrhythmias, chest pain, dizziness, or fainting in the last 3 months); (2) neuromuscular disease or orthopedic limitations; and (3) a self-reported active lifestyle (ie, patients achieving ≥150 min/week of moderate-intensity aerobic physical activity).

### Recruitment and Screening

**Figure 1:**
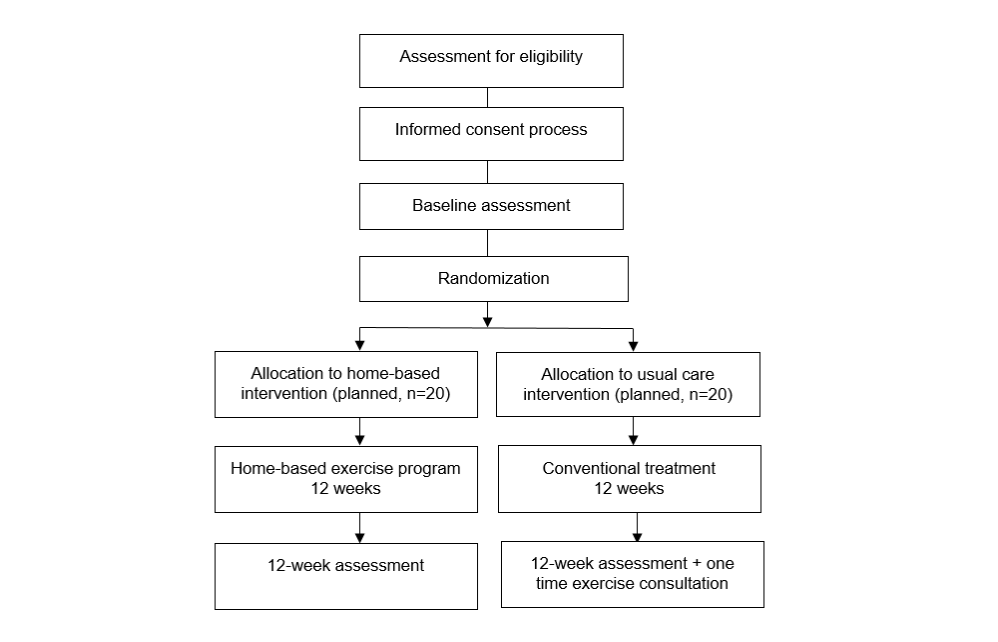
CONSORT (Consolidated Standards of Reporting Trials) flow diagram.

Potential participants will be recruited from posttransplant lung and liver clinics. The study team will identify potential participants at 12 to 18 months after transplantation with ≥2 metabolic risk factors. Participants will be asked by members of their circle of care if they are interested in participating in the study. If agreeable, the research team will contact the prospective participant to confirm eligibility criteria, discuss study participation, and conduct the informed consent process. The study flow of participants is shown in Figure 1.

### Randomization

After the baseline assessment, participants will be randomly allocated using a 1:1 ratio to either the intervention or the control group, stratified by transplant type (ie, liver or lung). The allocation of participants to their groups will be randomized using shuffled, opaque envelopes by a biostatistician with no involvement in data collection in the study. The research team, exercise professionals, and study participants will be aware of the group allocation.

### Study Assessments

#### Overview

Study assessments will be conducted at baseline and 2, 6, and 12 weeks. For a detailed list of assessment procedures by time point, refer to Tables 1 and 2. For participants unable to come on-site, assessments will be performed remotely from participants’ homes, and blood screenings and electrocardiogram assessments will be conducted at local laboratories. During remote assessments, participants will be supervised by the research team using videoconferencing. To ensure the safety and confidentiality of participants undergoing remote assessments, the research team will ascertain the participant’s identity, home address, and phone number. An assessment of the suitability of the participant to perform physical assessments will also be performed before starting any interaction with participants (eg, self-reported breathlessness, dizziness, and chest pain). Participants will be instructed to discontinue exercise testing if they experience any symptoms or feel unwell.

**Table 1:** Assessment of primary study outcomes by time point.

Primary outcomes	Assessment measures	Time point
Recruitment	Consented patients over total of eligible patients approached	During study period
Retention	Measured by attrition throughout the intervention period	During study period
Adherence	Completion of at least 70% of intervention elements	During study period
Satisfaction	Satisfaction survey	Weeks 2, 6, and 12^a^

^a^For usual care, satisfaction will be evaluated at baseline and 12 weeks.

**Table 2:** Assessment of secondary study outcomes by time point.

Secondary outcomes	Assessment measures	Time point
** Metabolic risk factors**		
Glucose tolerance (fasting glucose and HbA1c^a^ test), insulin sensitivity, (fasting insulin and HOMA-IR^b^), cholesterol panel, and C-reactive protein Diabetes, cholesterol, and hypertension medications Abdominal obesity	Blood work and medical charts Blood pressure monitor, charts, and home measurement	Baseline and 12 weeks
Liver fibrosis and steatosis (OLT^c^ patients attending assessments on-site)	Transient elastography (liver stiffness and controlled attenuation parameter for steatosis assessment)	Baseline and 12 weeks
**Clinical parameters**		
Model for end-stage liver disease at transplant Lung allocation score Medication history Electrocardiogram Liver enzymes Hospital admissions Graft function and comorbidities	Medical charts	Baseline and 12 weeks
Physical function	Short performance physical battery	Baseline and 12 weeks
Body composition	Bioelectrical impedance Waist circumference BMI	Baseline and 12 weeks
** Self-reported outcomes**		
Quality of life Barriers to exercise Physical activity behaviors Dietary habits	Short-Form Survey-36 Lifestyle questionnaire Physical Activity Scale for the Elderly Rapid Eating Assessment 3-day nutritional intake	Baseline and 12 weeks
Exercise self-efficacy	Exercise Self-efficacy Scale^d^	Baseline, 2, 6, and 12 weeks^d^

^a^HBA_1c_: hemoglobin A_1c_.

^b^HOMA-IR: homeostatic model assessment for insulin resistance.

^c^OLT: orthotopic liver transplant.

^d^For usual care, exercise self-efficacy will be evaluated at baseline and 12 weeks.

#### Study Arms

Following randomization, all study participants will be provided with a physical activity tracker (Fitbit) and exercise or physical activity logs to monitor exercise adherence and capture lifestyle changes in the intervention group and any contamination in the control group. Given the importance of diet on metabolic risk factors, both study groups will receive a counseling session on healthy eating consistent with the Canadian Food Guide by a registered dietitian, a review of the 3-day food record, and a nutritional handout. Nutritional advice will be tailored to specific metabolic concerns (eg, weight management, diabetes, and hypercholesterolemia). The dietitian will review all participants’ medical histories and pertinent bloodwork as routine care during dietary assessments.

#### Intervention Group

##### Overview

Our exercise program was informed by expert guidelines on exercise prescription in solid organ transplant recipients [12,20,41] and will be delivered by an exercise professional with experience in chronic disease management. Following the baseline assessment and randomization, intervention participants will receive an individualized, home-based exercise program consisting of aerobic and resistance training exercises for 12 weeks. The exercise prescription targets the Canadian Physical Activity guidelines [42], which recommends ≥150 minutes of at least moderate-intensity aerobic training per week and at least two days per week of resistance training for major muscle groups. Daily exercise practice will be unsupervised; however, the exercise professional will provide the exercise prescription during the first training session via videoconference, including the following: an assessment of the participant’s ability to exercise independently in their home environment (eg, review of the participants’ heart rate, blood pressure, and history of any pertinent injuries), demonstration and review of training techniques, and proper use of their Fitbit and exercise equipment.

Participants will also receive a detailed manual describing the importance of exercise and self-management skills for exercise behaviors based on the theory of planned behavior and social cognitive theory adapted from previous studies [43,44]. The manual will highlight the basics of exercise training, the importance of routines, principles related to exercise progression, and photographs with descriptions of the prescribed exercises. After the initial training session, the exercise professional will follow up weekly (via videoconference) with study participants to address potential barriers to exercise, provide motivational support, review symptoms or adverse events during independent training at home, and prescribe training progression. The exercise program will be tailored to the specific and changing needs of the participant, taking into consideration comorbidities, exercise capacity, physical function, and availability of training equipment. Details on exercise prescription for the intervention group are briefly provided and described in Table 3.

**Table 3:** Exercise prescription details for participants in the intervention group.

Modality and frequency	Intensity	Time and volume of exercise	Type of exercise	Progression
Aerobic; 3-5 days/week	Moderate to vigorous (target Borg RPE^a^: 12-14/20) 65% to 85% of age-predicted maximum heart rate	30 minutes Continuous or interval training ≥150 minutes of at least moderate-intensity aerobic training per week	Walking Running Alternating walking and running bouts Biking	Gradual progression of training duration (up to 60 min/session) Increase in walking and running speed or inclination If cycling, increase in revolutions per minute or resistance Increase in prescribed percentage of maximum heart rate
Resistance; 2 days/week	Target Borg RPE: 12-14/20	1-3 sets 6 exercises 10-12 repetitions	Resistance band exercises: Biceps curl Triceps extension Low row Shoulder abduction Body weight exercises: Wall squats Calf raises	Resistance intensity will be increased when 3 sets of 12 repetitions can be completed without difficulty to maintain moderate exertion Increase in resistance band tension Increase in the number of sets, reduction in resting time

^a^RPE: rating of perceived exertion.

##### Aerobic Training

The aerobic exercise prescription comprises three to five 30-minute sessions per week at a moderate intensity of 65% to 85% of the estimated maximal heart rate or 12-14/20 Borg rating of perceived exertion (RPE) scale [45]. The maximum heart rate will be estimated using the formula 207-(0.7×age in years) [46]. Training modalities may include walking outdoors, using a treadmill, cycling with a stationary bicycle, or other modalities available to the participant. The duration of the aerobic training session will be adjusted or divided (eg, 2 sessions of 15 minutes) if the participant has limitations because of comorbidities.

##### Resistance Training

Resistance training will be prescribed for at least two days per week and will focus on general conditioning comprising 6 to 10 exercises targeting the major muscle groups (1-3 sets of 8-12 repetitions). Prescribed resistance training exercises will include a combination of resistance bands with different tension and body-weight exercises. Resistance intensity will be increased when 3 sets of 12 repetitions can be completed without difficulty to maintain moderate exertion despite muscular adaptation (target Borg RPE=12-14/20) [43,44].

##### Safety Precautions for Remote Exercise Training

Participants will be instructed to discontinue exercise training if they experience any symptoms or feel unwell and will be strongly encouraged to immediately contact a member of the research team upon noticing any adverse events. Participants will be advised to reduce the intensity of their training if they reach ≥15 on the Borg RPE scale, if oxygen saturation is less than 85%, or if they experience any symptoms (eg, severe shortness of breath). If participants report chest pain or discomfort (ie, uncomfortable feeling of pressure, pain, squeezing, or heaviness in the chest spreading to the shoulder, arms, neck, and back), they will be instructed to stop and rest and seek emergency assistance if the discomfort persists for 5 minutes.

Participants who communicate medically concerning adverse events (eg, cardiovascular event or disease exacerbation) or contraindications to the exercise program will have their training suspended until clearance is provided by the study physician and the exercise professional. If participants sustain an injury because of participating in the study, the study team will ensure that appropriate medical treatment is received.

Before initiating any interaction with study participants, the study team will perform an objective assessment (ie, by measuring oxygen saturation, blood pressure, and heart rate if participants have a pulse oximeter or a blood pressure monitor at home). A subjective assessment of the study participants (eg, self-reported breathlessness, dizziness, and chest pain) will ascertain their suitability to proceed with testing, training, and progress with the exercise prescription. The study team will discuss with participants aspects of their home environment, availability and suitability of exercise training equipment, home walking space, and any potential safety concerns (eg, footwear, rugs, clutter, and pets). During the internet-based training sessions, participants will be supervised by the study team via a videoconference platform. Participants will be able to book additional sessions with the exercise professional if any concerns or questions arise.

#### Control Group

The control group will meet with the exercise professional at the start of the study to highlight the benefits of physical activity and will be provided with physical activity monitors (Fitbits) to track their daily physical activity levels (minutes and steps) along with physical activity logs to record daily physical activity. The study team will correspond with the control group every 2 to 4 weeks during the study period to see if any questions arise regarding the physical activity logs and trackers. After 12 weeks, the control group will have the option of receiving an exercise session with the exercise professional. During this session, study participants will receive general physical activity and exercise training recommendations and an exercise manual tailored to the needs of LTx and OLT recipients.

### Outcomes

A schedule of study outcome measurements is provided in Tables 1 and 2.

#### Primary Outcomes

##### Feasibility

The feasibility of a larger RCT to assess intervention efficacy (ie, phase 3 clinical trial) will be assessed through recruitment rates, program adherence, contamination, attrition, and safety at the end of the 12 weeks.

##### Recruitment

The recruitment rate will be defined as the proportion of randomized patients relative to all eligible patients approached for study participation. A consent rate of 30% or greater has been established as our criteria to determine study feasibility for future projects. The reasons for study nonparticipation will be collected.

##### Retention

Retention for the whole study and per group will be determined by the number of participants completing the final study assessment compared with the number of randomized participants. A retention rate of 80% or greater has been established as our criteria to determine study feasibility for future projects. The reasons for dropping out or interruptions to training will be collected.

##### Adherence

Adherence to the prescribed exercise program will be defined as completion of at least 70% of intervention components by the intervention group. This will be ascertained during weekly communication with participants and through a review of completed daily exercise logs, indicating exercise days, duration, intensity, and frequency of exercise sessions. Similarly, contamination in the control group will be assessed using physical activity logs.

##### Safety

The safety of the intervention will be monitored via daily logs (record of adverse events) and will be ascertained by the qualified exercise professional and reviewed by the principal investigator during weekly meetings.

##### Satisfaction

Satisfaction with the exercise program and other study interventions (eg, nutritional counseling) will be assessed at weeks 2 and 6 and at the end of the 12-week intervention via a satisfaction survey (multiple-choice questions) created by the research team. The control group will also complete a satisfaction survey in which participants will provide feedback on their experiences at the end of the study.

#### Secondary Outcomes

Preliminary efficacy data will be collected at baseline and 12-week assessments (or the last recorded day of program completion). Details on the study measures by time point are presented in Table 2.

##### Metabolic Risk Factors

Impaired glucose tolerance, abdominal obesity, cholesterol, and blood pressure will be assessed and classified according to the National Cholesterol Education Program Adult Treatment Panel III criteria [47]. More specifically, the following markers will be used to classify metabolic risks: (1) increased waist circumference (>102 cm [>40 in] for men, >88 cm [>35 in] for women); (2) elevated triglycerides of ≥1.7 mmol/L (≥150 mg/dL); (3) low levels of high-density lipoprotein: <1 mmol/L (40 mg/dL, male), <1.3 (50 mg/dL, female), or on cholesterol medication; (4) a resting blood pressure of ≥130/85 mm Hg or on hypertensive medication; and (5) fasting plasma glucose levels of 6.1 mmol/L (110 mg/dL) or on pharmacotherapy for diabetes. Participants will have the option of completing bloodwork either on-site or externally in a medical laboratory. Blood will be collected in a fasting state (no eating and drinking for 8 hours before testing; however, water is allowed) and includes a total cholesterol panel (ie, total cholesterol, triglycerides, low-density lipoprotein, and high-density lipoprotein), fasting blood glucose level, hemoglobin A_1c_ test, and C-reactive protein levels. Insulin resistance will be captured using the homeostatic model assessment for insulin resistance protocol (fasting insulin multiplied by fasting blood glucose). Finally, C-peptide levels will be analyzed in participants receiving exogenous insulin therapy.

##### Clinical Parameters

A liver transient elastography (FibroScan) assessment will be performed in liver transplant recipients to assess the degree of liver fibrosis (thickening and scarring of tissues) and steatosis (the amount of fat in liver tissue). Medical charts will be reviewed to collect data on age, sex, transplant type, the model for end-stage liver disease and lung allocation score, and detailed medication history through pharmacy records with cumulative and average daily doses of prednisone (mg/day) and calcineurin inhibitors, mTOR inhibitors, and antimetabolites (mycophenolate mofetil). Furthermore, liver enzymes along with total bilirubin, hemoglobin, and renal function (creatinine) levels will be abstracted from chart review. Data on posttransplant hospital admissions, allograft rejection, and cardiovascular comorbidities will also be ascertained from medical charts from the time of transplant to the end of the study period. Lifestyle factors, such as current alcohol consumption and smoking, will be ascertained from the chart review.

##### Physical Activity and Exercise Behaviors

Previous physical activity and exercise training habits will be collected at baseline by self-report (after discussion with the research team) and by completion of the Physical Activity Scale for the Elderly questionnaire [48], a short survey created to assess physical activity levels in older adults commonly applied in solid organ transplant recipients [9].

##### Physical Function

The short performance physical battery [49] test is used to assess gait, balance, and lower extremity performance. Participants will be asked to stand in one position holding their balance, rise from a chair 5 times, and walk for 4 m while being timed at their usual pace. For remote assessments (via videoconference), the balance test may be excluded if participants report a history of balance impairments or falls.

##### Body Composition

Weight, BMI, and body fat percentage will be measured via bioelectrical impedance analysis (Tanita, DC-430U) for participants coming on-site for study assessments; otherwise, participants will be asked to measure their weight at home if they have a scale. Participants will be asked to measure and record their body weight once a week. Height data will be obtained from the medical charts. For participants coming on-site for study assessments, waist circumference will be measured by the research team according to the World Health Organization standardized protocol. For participants completing the assessment remotely, waist circumference will be self-assessed using a measuring tape via videoconference under the direct supervision of the research team.

##### Nutritional Outcomes

The dietary habits of our study participants will be assessed using the Rapid Eating Assessment for Patients [50] questionnaire and a 3-day food record.

##### Health-Related Quality of Life

The 36-Item Short-Form Survey [51] will be used to measure mental and physical domains of quality of life in our participants (scores range from 0 to 100, with higher scores representing higher HRQL). The Short-Form 36 has been routinely applied in both OLT [30] and LTx recipients [52].

##### Self-efficacy

The Exercise Self-efficacy Scale [53], a Likert scale with 4-point rating in which participants rate their confidence levels with regard to carrying out regular physical activities and exercise, will be implemented to measure self-efficacy toward exercise.

##### Barriers to and Facilitators of Exercise

Familiarity and comfort levels surrounding technology, barriers to exercise, and evaluation of previous experience with exercise, especially within the home environment, will be captured using a questionnaire developed by our research team.

### Statistical Procedures

#### Sample Size

The sample size of 40 (10 per study group and transplant type) is in keeping with the recommendations for a pilot study to assess feasibility [54]. A power calculation was not performed for the secondary outcomes, as the main purpose of this study is to determine feasibility and obtain point estimates for a larger RCT. The liver transplant and LTx program at the University Health Network each has approximately 150 to 200 recipients per year. Furthermore, the rates of PTMS among LTx and OLT recipients range from 30% to 50%; thus, we anticipate an approximate eligible pool of 50 transplant recipients per year for each organ group. Our previous consent rates for rehabilitation studies in these populations have been approximately 30% to 50% [55,56].

#### Proposed Data Analyses

Descriptive statistics (means, SDs, or medians and IQRs) will be used for continuous variables, and frequencies will be used for categorical variables to summarize program adherence, participant satisfaction, contamination, attrition, and safety in the intervention group, and to compare any differences between LTx and OLT recipients across these parameters using 2-tailed *t* tests and tests of proportion. Estimates at baseline and 12 weeks within- and between-group will be reported using means and 95% CIs to derive effect estimates for sample size calculations in subsequent trials. Statistical analysis will be performed by a statistician who will be blinded to the group assignments. All eligible participants (N=40) will be included in the data analyses based on intention-to-treat, depending on the group to which they are randomized.

#### Data Management and Quality Assurance

Our research team is trained in the study requirements, measurement protocols, and has expertise in conducting physical assessments in solid organ transplant recipients. The research team has critically appraised the external peer-review comments from the Canadian Donation Transplant Research Program (Multimedia Appendix 1) and has incorporated them into the study protocol. Standard operating procedures have been generated for all the protocol elements. The accuracy of the data entered in our research database will be periodically audited by the research coordinator. The research team will attempt to have participants complete the entirety of the study assessments, and if there are incomplete data for a specific participant or missing responses to questionnaires, missing data will not be imputed. Participants will be closely monitored by the research team and the exercise professional to maximize study retention and assess exercise adherence rates during the 12-week intervention. Participants in the intervention group will be educated on the importance of adhering to the intervention, logging progress, and completing study assessments. The research team will send reminders to intervention participants for weekly meetings, and study assessments will be booked at convenient times for participants.

The study participants will have a random identifier generated with only the coded identifier attached to the participant data. The study data will be stored on a password-protected server following the currently established institutional and national research ethics and privacy guidelines. The study information will be accessible only to approved study members. In the event of an inappropriate release of personal health information to an unauthorized party, we will take the appropriate steps to minimize any potential harm: (1) further release of information will be stopped, (2) an attempt to retrieve all inappropriately released information will be made, and (3) the sponsor’s privacy office and research ethics board will be immediately notified.

## Results

This research was funded in July 2020, and enrollment began in July 2021. It is estimated that the study period will be 18 months and that study assessment and data collection will be completed by December 2022. The study results will be submitted for publication in the first half of 2023.

## Discussion

### Overview

PTMS is a common sequela of solid organ transplantation and is associated with increased cardiovascular morbidity and mortality among LTx and OLT recipients [57,58]. Studies in the early posttransplant period (<1 year) have shown the benefits of facility-based exercise training on physical function and HRQL, but to date, only a recent study has evaluated the effects of 3 months of telehealth sessions on metabolic risk factors in OLT recipients [32]. These teleconferencing group sessions comprised alternating exercise and nutrition-based interventions with a few personalized sessions, which demonstrated improvement in PTMS, diet adherence, and mental HRQL. However, it remains unclear whether home-based exercise programs can be delivered with a sufficient exercise dose and adequate adherence to have effects on metabolic risk factors and exercise self-efficacy in both LTx and OLT recipients. This is an important consideration given the significant differences in routine perioperative rehabilitation practices across solid organ transplant recipients. For instance, LTx recipients participate in both pre- and posttransplant rehabilitation [41], whereas many centers do not have a dedicated exercise program for OLT recipients and may impact self-efficacy, adherence, and perception related to exercise training [12,59]. Thus, this protocol outlines the methodology of a pilot study implementing a partly supervised personalized home-based exercise program for LTx and OLT recipients as the first key step in developing future clinical trials to offset the high morbidity associated with PTMS.

### Home-Based Exercise Training

With fewer patients attending nonessential visits to health care centers during the COVID-19 pandemic, providing patients with tools to facilitate independent training has become a priority. There is increasing evidence that remote exercise interventions across a range of clinical populations have emerged in the last year [60-62], but more importantly in LTx and OLT recipients, showing promising results in training volumes and HRQL, respectively [26,32]. Furthermore, home-based interventions have become an important strategy in a number of chronic conditions and have the potential to be incorporated into the standard of care treatment plan for LTx and OLT recipients [63]. The novelty of the present home-based program lies in the promotion of exercise behaviors by increasing patients’ self-efficacy to exercise in a familiar environment with minimal equipment, resources, and supervision. Furthermore, home-based exercise training allows for reduced travel and increased convenience for participants to exercise in the comfort of their homes [26,32,64].

### Strengths and Limitations

Our study protocol has several strengths. First, we aimed to design a standardized, high-quality intervention grounded on theoretical and practical considerations specifically tailored for LTx and OLT recipients. The 12-18–month posttransplant period was chosen given the high rates of PTMS at this time point and the barriers experienced by patients [9,10]. We acknowledge that implementation of an exercise rehabilitation program early in the posttransplant period (eg, 3 months) may help prevent the occurrence of some of these metabolic risk factors, but we are specifically interested in addressing the knowledge gap of whether PTMS may be attenuated with a home-based exercise program in the late posttransplant period. Second, the home-based delivery format of this intervention constitutes a novel approach in the LTx and OLT populations. We prioritized implementation feasibility in the design of our protocol by creating an exercise program that can be replicated at home with minimal equipment and supervision while meeting safety parameters. Our intervention protocol was also designed to promote the sustainability of behaviors after the study period with exercises and activities that can be performed every day, such as walking, which also promotes transference to daily routines. Other highlights of our research include the inclusion of a control group, the rigor in our methodology (detailed and accurate reporting of methods according to the CONSORT, SPIRIT, and the Consensus on Exercise Reporting Template statements), and the inclusion of nutritional counseling for all study participants. Despite our trial being focused on exercise, we also acknowledge the importance of nutritional support in managing PTMS and made available a consultation with a dietitian as part of the educational component of our research.

We would also like to acknowledge the potential limitations of the study methodology. Given the complex profile of some of the patients diagnosed with PTMS, a 12-week intervention may not be sufficient to elicit a noticeable improvement in the metabolic profile of these patients. An extended intervention may have a greater impact on PTMS risk factors, insulin resistance, and physical function and will be considered in a future RCT. There is a potential risk of bias in our research given the lack of blinding of the exercise professional and study participants (double-blinding is often not achievable in exercise intervention studies) [65]. However, our study will aim to satisfy other aspects of the Revised Cochrane risk-of-bias tool for randomized trials [66]. Furthermore, we excluded patients diagnosed with unstable cardiovascular or neuromuscular disease to ensure participant safety and understand that this may reduce some of the generalizability across transplant recipients. We are also aware of the challenges to exercise training in this population (eg, osteoporosis and diabetes mellitus), and although we designed an exercise protocol achievable by most transplant patients, personalized adaptations of the intervention cannot be avoided and represent a key feature of our protocol. Finally, to facilitate the assessment of patients during the COVID-19 pandemic, we took steps to adopt our assessment protocol to provide participants with the possibility of performing study assessments from the participant’s home environment and bloodwork locally at community laboratories. We would like to acknowledge the potential threat to consistency in our assessment protocols, given that some assessments may be conducted on-site, whereas others will be performed remotely in the participant’s home.

### Conclusions

Exercise has become an important pillar in the management of comorbidities associated with LTx and OLT. A home-based exercise program may prove to be an effective posttransplant strategy for improving physical function and the metabolic profile of transplant recipients. Characterizing the feasibility, adherence, and effect estimates of home-based exercise training constitutes the first step in the promotion of a healthy lifestyle in transplant recipients and the establishment of long-term sustained change. This will be the first study to investigate the effects of exercise on PTMS risk factors, self-efficacy, and HRQL. The results of this trial can provide a greater understanding of behavioral strategies aimed at increasing exercise and physical activity in LTx and OLT recipients at risk of PTMS. Given the lack of patient education resources in the LTx and OLT populations, we hope that our results will provide greater insights regarding patients’ exercise preferences and exercise modalities to create patient-directed materials aimed at promoting healthy living after transplantation. Our research will create opportunities for future collaborations and initiatives to gain greater insights into the mechanisms of PTMS and reduce its high prevalence through lifestyle interventions.

## Appendix

Multimedia Appendix 1 Peer-review report from the Canadian Donation Transplant Research Program competition.

## Abbreviations

CONSORT: Consolidated Standards of Reporting Trials
HRQL: health-related quality of life
LTx: lung transplant
OLT: orthotopic liver transplant
PTMS: posttransplant metabolic syndrome
RCT: randomized controlled trial
RPE: rating of perceived exertion
SPIRIT: Standard Protocol Items: Recommendations for Interventional Trials
