# Supplementary material for: Feasibility of a Home-Based Exercise Program for Managing Posttransplant Metabolic Syndrome in Lung and Liver Transplant Recipients: Protocol for a Pilot Randomized Controlled Trial
Source: JMIR Res Protoc. 2022 Mar 23;11(3):e35700. doi: 10.2196/35700 (PMC8987959; doi:10.2196/35700)
Supplement: Multimedia Appendix 1 [file resprot_v11i3e35700_app1.pdf]

The Canadian **DONATION** and **TRANSPLANTATION** Research Program
Programme de recherche en **DON** et **TRANSPLANTATION** du Canada

COMMENTS & SCORES FROM COMMITTEE MEMBERS

2019 CDTRP Research Innovation Grant Competition - Peer Review

Applicant Name: Dmitry Rozenberg
Grant Title: Feasibility of a Home-Based Exercise Program in Liver and Lung Transplant Recipients for Management of Post-Transplant Metabolic Syndrome: A Pilot Randomized Controlled Trial

Total number of eligible applications submitted to the competition: 47
Final committee score (out of 5.0): 4.37
Final ranking: 2 out of 47
Number of grants funded in this competition: 18

Comments from Reviewer #1:

A brief synopsis of the proposal
Purpose: To evaluate the feasibility of a three-month individualized, digitalized home-based aerobic and resistance training program in OTL and LTx recipients starting at 1-year post-transplant and assess its efficacy
HYPOTHESES: We hypothesize that it will be feasible to recruit both OLT and LTx recipients into a homebased exercise program with ≥ 75% adherence to the prescribed exercise dose.
Design: pilot, clustered RCT

An assessment of the proposal, based on the evaluation criteria as presented in the funding opportunity details:
This is a well written proposal and authors have sufficiently summarized the background, their plan and goals moving forward.
It is a feasibility study
The exact age group they are targeting could be mentioned; ideally it should be a middle aged group
Integration of patient partners is not well-defined in the protocol (although a little bit is mentioned in the integration section).

Comments on the budget requested and a formal recommendation
Well described
How is the nutritionist supported?

Comments on the proposed integration with the CDTRP and how it will contribute to the mandate of the CDTRP
Well integrated with theme 5

If necessary, comments on issues the reviewer feels should be flagged.

Comments from Reviewer #2:

A brief synopsis of the proposal
A pilot, clustered RCT of an individualized home-based exercise versus usual care in 20 orthotopic liver transplant (OLT) and 20 lung transplant (LTx) recipients with post-transplant metabolic syndrome (PTMS) at one-year post-transplant. The aims of the study are: 1) To evaluate the feasibility (recruitment rate, program adherence, attrition, safety, and participant satisfaction) of a three-month individualized, digitalized home-based aerobic and resistance training program in OTL and LTx recipients starting at 1-year post-transplant, and **2)** To assess estimates of intervention efficacy on elements of PTMS, insulin resistance, exercise self-efficacy, and quality of life. This study is a key step in the development of future clinical trials designed to reduce the high morbidity associated with PTMS.

An assessment of the proposal, based on the evaluation criteria as presented in the funding opportunity details:
• The grant application is well written (both clear and concise) with the proposed objectives in alignment with those of the funding opportunity
• The proposal clearly articulates the rationale for pursuing the proposed research
• Appropriate research design and methodology
• The research seems feasible, with reasonable potential for clinical impact
• The novelty of this research is its focus on promoting exercise adherence through strengthening self-efficacy and utilizing standard health technology, which has not been applied in transplant studies to-date.
• This will be the first study to investigate the effects of exercise on PTMS, metabolic risk factors and insulin resistance. Characterizing the feasibility, adherence, and effect estimates of home-based exercise training constitutes the first key step in promoting a healthy lifestyle in transplant recipients, and supporting the development of future trials aimed at reducing the morbidity associated with PTMS
• The research team is an established collaboration of multi-disciplinary clinicians and investigators. PI is an early career investigator
• Would benefit from a more innovative KT plan

Comments on the budget requested and a formal recommendation
• Budget appears appropriate and well justified. Inclusion of honorariums for patient partners.

Comments on the proposed integration with the CDTRP and how it will contribute to the mandate of the CDTRP

• Research is well aligned with Theme 5 and will create novel opportunities for future collaboration with other investigators as part of Theme 5 – CAN-RESTORE

Scientific officer notes:

The protocol is well written and concise. The work proposed is aligned with the competition. The investigator is early in career. There is significant potential for any findings arising from the proposed research. The knowledge translation section was not well developed and for future grants to this and other granting agencies, more development and detail would be recommended.
